# State and trait of finger tapping performance in multiple sclerosis

**DOI:** 10.1038/s41598-021-96485-3

**Published:** 2021-08-24

**Authors:** Philipp Gulde, Heike Vojta, Joachim Hermsdörfer, Peter Rieckmann

**Affiliations:** 1 Centre for Clinical Neuroplasticity, Medical Park Loipl (Medical Park Group), Thanngasse 15, 83483 Bischofswiesen, Germany; 2 Technical University of Munich, Munich, Germany; 3 Friedich-Alexander University Erlangen-Nurnberg, Erlangen, Germany

**Keywords:** Neurological disorders, Human behaviour, Disability, Fatigue, Multiple sclerosis

## Abstract

Finger tapping tests have been shown feasible to assess motor performance in multiple sclerosis (MS) and were observed to be strongly associated with the estimated clinical severity of the disease. Therefore, tapping tests could be an adequate tool to assess disease status in MS. In this study we examined potential influencing factors on a maximum tapping task with the whole upper-limb for 10 s in 40 MS patients using linear mixed effects modelling. Patients were tested in three sessions with two trials per body-side per session over the course of 4–27 days of inpatient rehabilitation. Tested factors were the expanded disability scale (EDSS) score, laterality of MS, age, sex, hand dominance, time of day, session, trial (first or second), time between sessions, and the reported day form. A second model used these factors to examine the self-reported day form of patients. Linear mixed effects modelling indicated the tapping test to have a good inter-trial (proportional variance < 0.01) and inter-session reliability (non-significant; when controlling for time between sessions), an influence of hand-dominance (proportional variance 0.08), to be strongly associated with the EDSS (eta^2^ = 0.22, interaction with laterality of MS eta^2^ = 0.12) and to be not associated with the reported day form. The model explained 87% (p < 0.01) of variance in tapping performance. Lastly, we were able to observe a positive effect of neurologic inpatient rehabilitation on task performance obvious from a significant effect of the time between sessions (eta^2^ = 0.007; longer time spans between sessions were associated with higher increments in performance). Day form was only impacted by EDSS and the time of the day (p < 0.01, R^2^ = 0.57, eta^2^_TIME_ = 0.017, eta^2^_EDSS_ = 01.19). We conclude that the tapping test is a reliable and valid assessment tool for MS.

## Introduction

Multiple sclerosis (MS) is an autoimmune mediated, chronic, neurological disease that affects the central nervous system, with a prevalence of approx. 1–2 in 1000 persons in central Europe^1,2^. The common onset is in early adulthood and a plethora of different symptoms can arise from a commonly initially relapsing immune reaction against the myelin sheath of neurons of the central nervous system^3^. Motor symptoms can be a reduced muscle strength, spasticity, an increased fatigability, and coordinative impairments like ataxia^4–7^. Additionally, patients with MS often report strong variations due to their current state, e.g. concerning the experienced fatigue^8^ or vision^9^.

Finger-tapping tasks have been observed to be able to give estimates on central excitability and conductivity in stroke^10^ and healthy individuals^11^ and to be good markers of disease progression in MS^12–14^. Therefore, tapping tasks could be an adequate measure in cross-sectional and longitudinal examinations (or studies) to quickly assess disease progression (or regression) as well as impact of medication in a sensitive manner.

**Figure 1:**
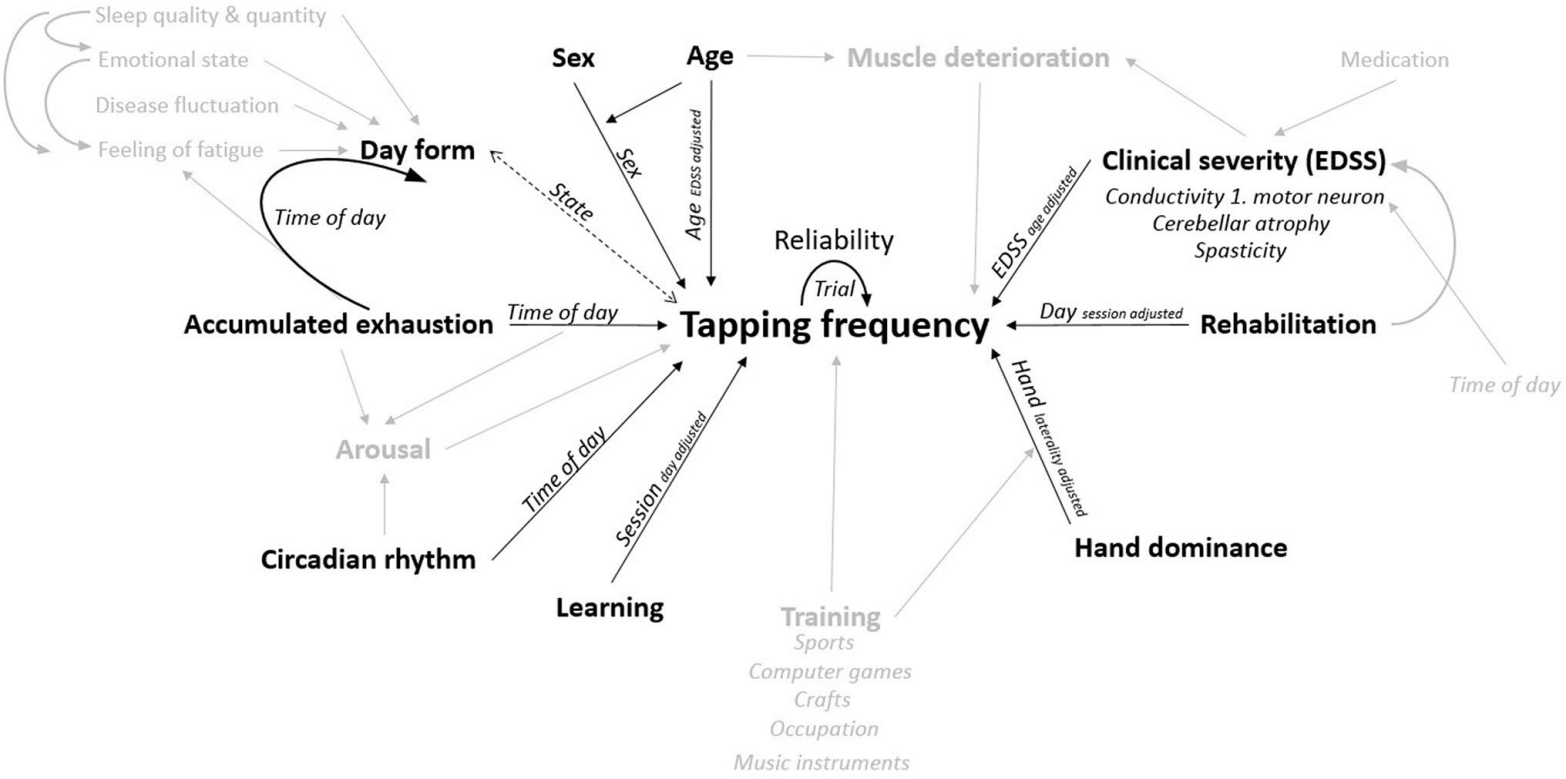
Effect model of factors influencing free finger tapping performance based on current literature. Factors that are in grey were not examined in this study.

The current literature suggests the following factors to be influencing the tapping frequency in healthy adults and neurologic patients (Fig. 1): Age and sex (with a stronger age-related decline of performance in women)^15–17^ and hand-dominance^15^ show an impact on the tapping frequency. Further, learning the tapping task can increase the tapping frequency^11,18^, same as transfer of training of sports, computer games, crafts, occupation (as education), and playing music instruments^17^. Newsome et al. observed that finger tapping frequencies were reduced in higher (> 4.0) expanded disability scale scores (EDSS)^19^. They further found a relationship between grip and pinch strength and tapping frequency, but not for EDSS and grip strength (cross-sectional), which could indicate a secondary deterioration of muscle mass (reduction of muscle mass by lifestyle and not primarily disease). Gulde et al.^20^ described a strong association between finger tapping frequencies (sum of dominant and non-dominant upper-limb) and EDSS. Another factor is the time of the day, which could negatively impact tapping performance by an accumulated exhaustion that is often reported in MS^21^ or in both directions by the individual circadian rhythm^22–24^ or body core temperatures (e.g., after physical activity)^25^. Additionally, arousal, for instance after physical activity or due to exhaustion, can influence the tapping frequency^22^. It has been shown that fatigue (in chronic fatigue patients) does not negatively influence finger tapping rates^26^ and that motor performance and the feeling of fatigue appear to be not associated^21,27^. On the other hand, the daily form (the felt day-state as in “Today, I will beat my personal best.”), estimated by the subjects, can bias motor-performance^28^ (or the interpretation of it). Wurtele^29^ reviewed a very weak association between motor performance (in golf-putting) and self-efficacy (controlling for prior task performance). Finally, and to point on the remaining factor “rehabilitation” (Fig. 1), the effects of physical exercise, which is a core component of rehabilitation in neurology, on finger tapping have been reported to be beneficial (yoga)^30^ as well as inpatient neurorehabilitation^20^.

In this study, we wanted to estimate the partial variance that can be explained by session, trial, time of day, hand-dominance, age, sex, and severity of MS (EDSS) in a finger-tapping task. All covered factors are summarized in Table 1. Further, we tested, if patients could estimate their day form (state). We hypothesized that the felt “current form” (state) by patients is only partially associated^29^ with the measured performance and that finger-tapping task performance is therefore stable (a “sensorimotor trait”) and reliable when assessing MS patients’ performance.

**Table 1:** Effects covered in the current study.

Factor	Short explanation	References
Clinical severity (EDSS)	Higher disability can lead to worse performance	^19,20^
Rehabilitation	Rehabilitation can improve performance	^20,30^
Age, sex and age × sex	Worse performance in advanced age, especially in women	^15–17^
Hand dominance	Better performance when using the dominant hand	^15^
Day form	Potential emotional bias that could influence performance	^28^
Accumulated exhaustion	Accumulated exhaustion can negatively impact performance	^21^
Circadian rhythm	Performance can be influenced by the circadian rhythm and therefore by the individual time of day	^22–24^
Learning	Learning and training can improve performance	^11,17,18^
Reliability	Reliability of the test. Does not influence performance	–

## Methods

### Sample

A convenience sample of 40 MS inpatients were recruited at the Center for clinical Neuroplasticity, Medical Park Loipl (Medical Park Group), a specialist clinic for neurology in Germany. Sample characteristics are given in Table 2. Inclusion criteria were the ability to give informed consent (including a minimum age of 18a) and a diagnosed MS. Exclusion criteria were orthopedic or neurological comorbidities and being in an acute to sub-acute relapse state. All participants gave written informed consent to participate in the “*Implementation of a Neuro Assessment Lab*”* project*. Ethical approval was given by the ethics committee of the Medical Faculty of Technical University of Munich. The experiment was conducted in accordance with the applicable relevant guidelines and regulations.

**Table 2:** Sample characteristics: ratios, means, standard deviations, and ranges.

n	Sex	Hand dominance	Laterality of MS MS type		Age	EDSS	Days first to last session
40	65% female 35% male	95% right 5% left	42.5% dominant side 57.5% non-dom. Side 60% RR (24/40) 40% PMS (16/40)	Mean STD Median Range	49.2a ± 10.3a 52a 23–63a	4.0 ± 2.0 3.75 1.0–8.0	17.4d ± 6.2d 19.5d 4–26d

*RR* relapsing remitting MS, *PMS* progressive MS.

All patients followed a scheduled inpatient rehabilitation program covered by health and pension insurances. Therapies followed international guidelines and emphasized physio- and occupational therapy (approx. 85% of scheduled sessions).

### Procedure

The tapping frequency of the dominant and non-dominant hand was assessed in sitting-position twice on three different dates for a duration of 10 s per trial (in the following order: dominant, non-dominant, dominant, non-dominant, without extended breaks between trials). Time spans between and time points of sessions was dependent on the participants’ schedules during their stay. Session 2 was in average 7 days after session 1, and session 3 in average 11 days after session 2. The range of time spans between first and last session was 26 days. Patients were allowed to use their complete upper-limb to execute the task and were instructed to tap as fast as possible: ‘Please tap as fast as possible with your fingertip on the target on the smartphone screen until the target disappears after 10 s. You are allowed to use your whole upper-limb. You are allowed to start at any time.’ (translated from German). Each contact of the fingertip on a smartphone screen (via a red-colored digital button of 5.5 cm in width and 5.8 cm in height; the Lumia550 (Microsoft Cooperation) smartphone laying on the surface of a desk) was recorded, starting to record with the first contact. Patients were allowed to use their whole upper-limb in the most efficient way in order to allow persons with higher levels of disability to still perform the task. We assumed that patients automatically used the most efficient movement strategy. A prior study from our workgroup has shown that this task is a valid instrument to assess sensorimotor control in multiple sclerosis^20^. The duration of 10 s was used in studies examining stroke^10^ and Parkinson’s disease^12^ patients. The used smartphone application was customized and already used in prior work^20^.

Further, on each session, patients were asked to score their “current form” (German: “Tagesform”–“day state”) on a scale from 1 to 6 with 1 being the best possible state (rating as in the German school system). We recorded the following parameters:

- Tapping frequency (**TAP**) as main outcome in [Hz]
- Trial (**TRIAL**), either 1 or 2
- Session (**SESSION**), 1, 2, or 3
- Hand (**HAND**), the used hand: either dominant or non-dominant
- Stronger impaired hand (laterality: **LAT**), either 0 (*yes*) or 1 (*no*)
- Sex (**SEX**), either 0 (*male*) or 1 (*female*)

As further factors, we used:

- Time of the day (**TIME**) in [h] (e.g., 12:30 = 12.5)
- “Current form” (day state) (**STATE**)^1^
- Age (**AGE**) in [a]
- EDSS (**EDSS**)
- Day since first session (**DAY**), with the first session being day = 1

^1^**1**: very good, **2**: good, **3**: average/satisfactory, **4**: sufficient, **5**: not sufficient, **6**: poor; commonly treated as a continuous variable as it is generally used in the German education system

### Statistical analysis

We used the *lme4*^31^ and *lmerTest*^32^ RStudio packages for linear mixed effects modelling for the following initial response function^33^:

$$ \begin{aligned} & {\text{TAP }}\sim { 1}\left| {{\text{TRIAL }} + { 1}} \right|{\text{SESSION }} + { 1}\left| {{\text{HAND }} + { 1}} \right|{\text{SUBJECT }} + { 1}\left| {{\text{LAT }} + { 1}} \right|{\text{SEX }} + {\text{ TIME }} + {\text{ STATE }} \\ & \quad + {\text{ AGE }} + {\text{ EDSS }} + {\text{ DAY }} + {\text{ TIME}}:{\text{STATE }} + {\text{ STATE}}:{\text{EDSS }} + {\text{ TIME}}:{\text{EDSS }} + {\text{ LAT}}:{\text{EDSS,}} \\ \end{aligned} $$

where 1|X denotes a random effect and X:Y an interaction term.

Random and fixed effects were used following the description by Ramsey and Schafer^34^, with fixed and random group means as the defining characteristics.

Trial, session, hand, sex, and laterality were used as random effects, time, state, age, day, and EDSS as fixed effects. Interactions between time and state, state and EDSS, and time and EDSS (fixed effects) were anticipated. The formula was adapted using a backwards selection (significance as criterion)^32^. Further, a second model was calculated for STATE. STATE was treated as a continuous variable with low resolution. In order to circumvent ties in the STATE model, we additionally overlaid uniformly distributed noise of − 0.5 to + 0.5 to STATE over 10.000 iterations to give credible and confidence intervals of the significance levels of the model’s factors. Homogeneity of variance in TAP was examined for the first session for SEX, HAND, TRIAL, and LAT, and for SESSION by Fligner–Killeen’s tests. Further, TAP and STATE were checked for normal distribution by a chi-squared test due to data ties (but no estimate of rounding difference^35^). A post-hoc estimation of statistical power for the TAP model was computed by a simulation with 1000 iterations based on the intercept, coefficients (fixed effects), and variance (random effects) of our data. For each iteration, analyses of variance comparing the models with and without the respective factors were run. Power was given by the probability of p < 0.05.

Additional bivariate correlations between the mean tapping frequency in the first session and the EDSS, as well as between tapping frequency and (adjusted) reported day forms were computed in order to display the practicability and robustness of the tapping test. Variance inflation (VIF) was set to VIF < 5.0. α was set to 0.05. Statistics were run in RStudio (RStudio Inc.).

## Results

### Descriptives

**Figure 2:**
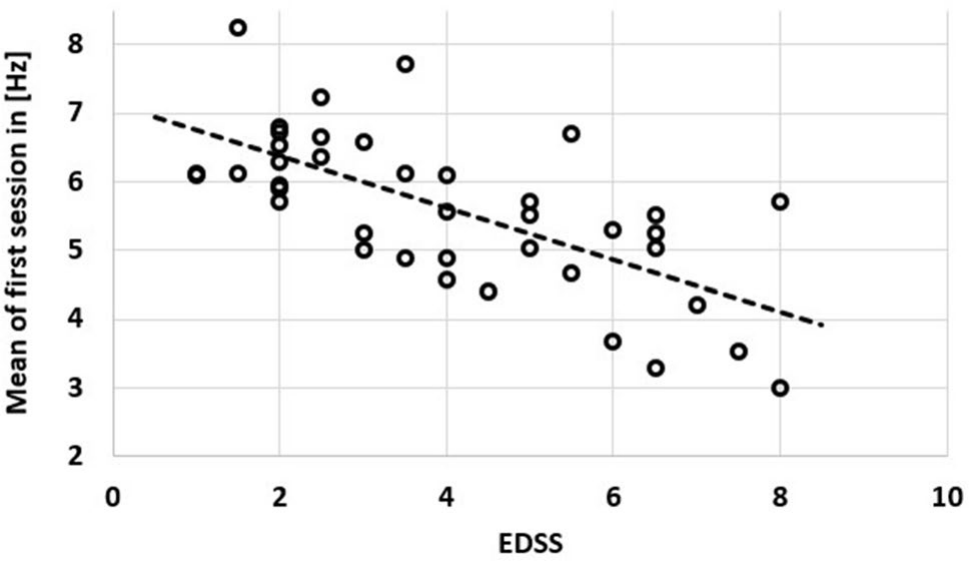
Scatter plot of EDSS and the mean of both hands and trials of the first session of finger tapping. The association was strong with an R^2^ of 0.45 (p < 0.01).

Participants revealed quite stable TAP (Table 3, TRIAL had 0.5% proportional variance, DAY 0.7% and SESSION was non-significant) and reported comparable STATE (Table 3, no significant impact of DAY or SESSION on STATE) over the three sessions during the rehabilitation program (Table 3). STATE was overall rated as *average* (according to the grading system). The mean (both hands) tapping frequency in the first session was strongly associated with the EDSS (Fig. 2) with an R^2^ of 0.45 (p < 0.01, R^2^_CI95_ = [0.21, 0.66]; the mean was chosen to better display global and body-side specific impairments) (dominant hand: R^2^ = 0.49, p < 0.01, R^2^_CI95_ = [0.25, 0.69]; non-dominant hand: R^2^ = 0.32, p < 0.01, R^2^_CI95_ = [0.09, 0.55]; hand on stronger impaired body-side: R^2^ = 0.46, p < 0.01, R^2^_CI95_ = [0.22, 0.67]; hand on not stronger impaired body-side: R^2^ = 0.32, p < 0.01, R^2^_CI95_ = [0.09, 0.55]).

**Table 3:** TAP, DAY, and STATE on the three session.

Session	TAP in (Hz)	DAY in (days)	STATE
1	5.60 ± 1.15	1 ± 0	3.05 ± 1.20
2	5.62 ± 1.18	8.5 ± 5.7	3.18 ± 1.22
3	5.74 ± 1.21	18.4 ± 6.2	3.08 ± 1.16

Homogeneity of variance was given for TAP by SEX (p = 0.11), HAND (p = 0.98), TRIAL (p = 0.86), and LAT (p = 0.13), and for SESSION (p = 0.91). TAP and STATE were assumed as being normal distributed with a p-value of 0.40 for TAP and p = 0.47 for STATE.

### Modelling: TAP

Linear mixed effects modelling resulted in the following formula:

$$ {\text{TAP }}\sim \, \left( {{1}|{\text{TRIAL}}} \right) \, + \, \left( {{1}|{\text{SUBJECT}}} \right) \, + \, \left( {{1}|{\text{HAND}}} \right) \, + {\text{ DAY }} + {\text{ EDSS }} + {\text{ LAT}}:{\text{EDSS}}{.} $$

TAP was best explained by the random effects TRIAL, SUBJECT, and HAND and the fixed effects DAY and EDSS with an interaction of LAT and EDSS.

**Figure 3:**
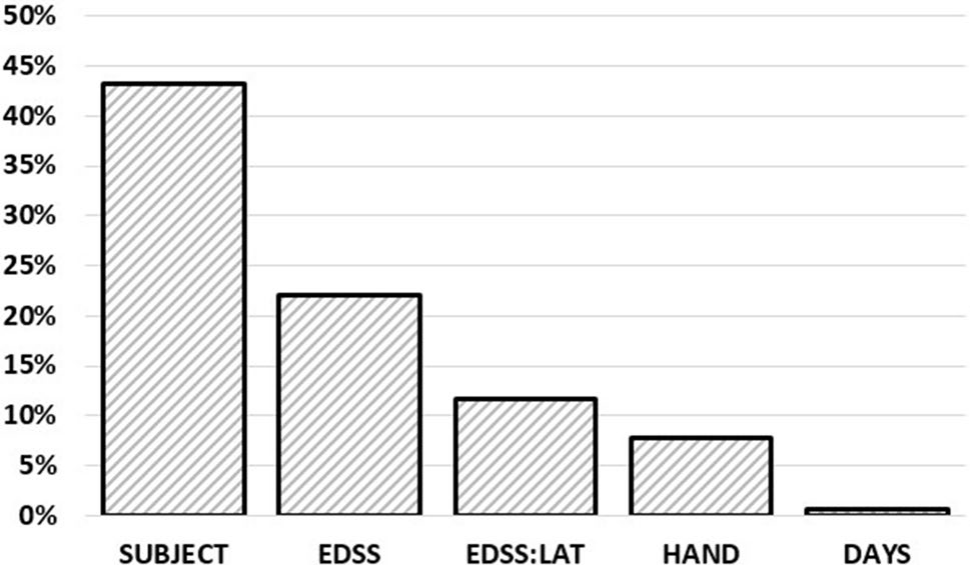
Comparison of explained variance (SUBJECT, TRIAL, HAND) and eta^2^ estimations (DAYS, EDSS, EDSS:LAT).

**Figure 4:**
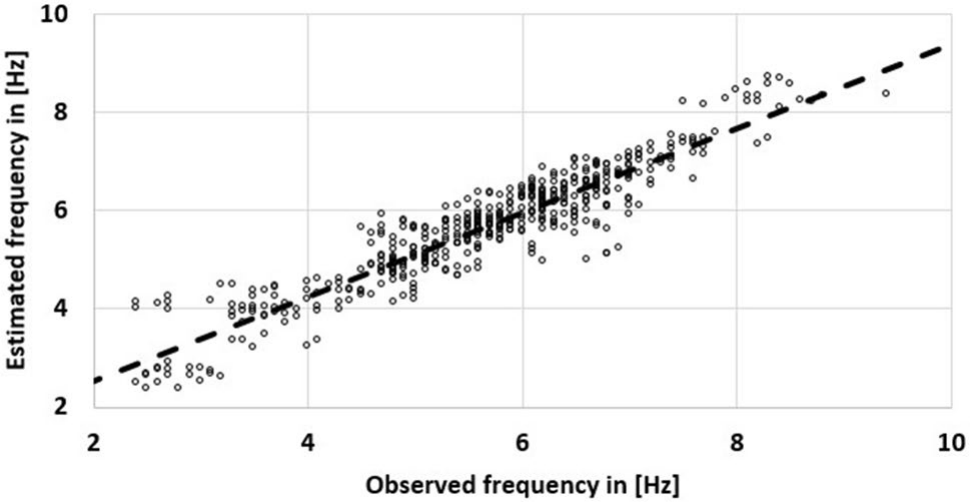
Observed and estimated tapping frequencies in (Hz) for a total of 480 data points (40 subjects × 3 sessions × 2 trials × 2 hands). The regression has an explained variance of 0.87 (p < 0.01).

The included factors are given in Table 4. The effects of TRIAL and DAY were minimal, while HAND and LAT:EDSS reveled moderate effects. The slopes of the fixed effects were EDSS: − 0.3305 Hz/points and DAY: 0.0063 Hz/day. A comparison of explained variance and estimated eta^2^ are illustrated in Fig. 3. The resulting estimates of TAP and the observed tapping frequencies were strongly associated with an R^2^ of 0.87 (p < 0.01, R^2^_CI95_ = [0.85, 0.89], Fig. 4).

**Table 4:** Characteristics of the linear-mixed effects model.

	Factor	p as level of significance	Proportional explained variance	Effect size in Cohen’s d	Variance inflation factor
Random effects	SUBJECT	< 0.01	0.43	–	–
	TRIAL	0.015	0.005	–	–
	HAND	< 0.01	0.08	–	–
	RESIDUAL	–	0.14	–	–
Fixed effects	DAY	0.026	0.007 (eta^2^)	0.21	1.00
	EDSS	< 0.01	0.22 (eta^2^)	1.56	1.00
	LAT:EDSS	< 0.01	0.12 (eta^2^)	1.00	1.00

Post-hoc power analyses resulted in powers of EDSS: 0.974, HAND: 0.812, TRIAL: 0.333, LAT:EDSS: 0.060, DAY: 0.033.

### Modelling: STATE

Linear mixed effects modelling resulted in the following formula:

$$ {\text{STATE }}\sim {\text{ TIME }} + {\text{ EDSS }} + \, \left( {{1}|{\text{SUBJECT}}} \right). $$

SUBJECT had an explained variance of 0.57 (p < 0.01), the estimated proportional eta^2^s for TIME and EDSS were 0.017 (TIME, p < 0.01, d = 0.31, variance inflation factor = 1.00) and 0.19 (EDSS, p < 0.01, d = 1.24, variance inflation factor = 1.00). The slopes were 0.273/points for EDSS and 0.055/h for TIME.

The level of significance over 10.000 iterations resulted in SUBJECT: p_mean_ =  < 0.00001 CI_95_ = [< 0.00001; < 0.00001] credible interval_95_ = [< 0.00001; < 0.000001], TIME: p_mean_ = 0.00701 CI_95_ = [0.00659; 0.00705] credible interval_95_ = [0.00013; 0.03674], EDSS: p_mean_ = 0.00054 CI_95_ = [0.00053; 0.00054] credible interval_95_ = [0.00027; 0.00092].

**Figure 5:**
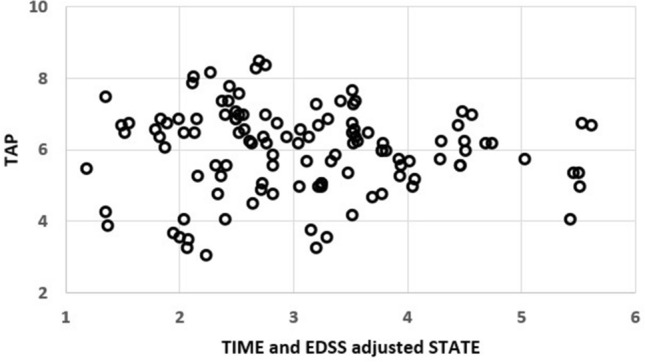
Scatter plot of TAP and TIME and EDSS adjusted STATE (R^2^ < 0.01, p = 0.54).

After adjustment for TIME and EDSS, TAP and STATE were not associated (Fig. 5, R^2^ < 0.01, p = 0.54).

## Discussion

In this study, we assessed the finger tapping frequency (10 s trial duration with free use of the whole upper-limb) in 40 MS patients over the course of a neurologic inpatient rehabilitation. Linear mixed effects modelling revealed a set of significant factors predicting performance. A second model, examining the patient reported “current form” (day state) comprised solely the time of the day and the clinical severity of MS (as EDSS).

The model for TAP showed a good inter-trial reliability with a variance proportion of only 0.005 for TRIAL. While potential effects of TIME (accumulated exhaustion, circadian rhythm, Fig. 1), SESSION (learning, Fig. 1), STATE (day form, Fig. 1), SEX (biological sex, Fig. 1), and AGE (age, Fig. 1; as well as a potential interaction of sex and age) were too weak to be included in the model, other factors revealed weak to strong impact. The missing impacts of TRIAL and SESSION suggest that participants either automatically choose the most efficient movement strategy or at least showed no tendency to change their movement strategy (changes of strategy between trials could have been beneficial or detrimental, however, beneficial changes of strategy would have been transferred to the next session). TAP was impacted by hand dominance (HAND), global clinical severity of MS (EDSS), body-side specific emphasis of MS (LAT:EDSS as laterality of MS symptom severity), and rehabilitation (DAY). The tapping tasks proved to be a valid (EDSS and LAT:EDSS) and reliable assessment tool due to its good inter-trial (TRIAL; two trials for each hand on three occasions) and inter-session (SESSION) reliability and its strong association with the clinical severity of MS (Fig. 2), globally (EDSS) and specifically (EDSS:LAT). The effects of rehabilitation appeared to be relatively small, although, when setting the slopes for EDSS (− 0.3305 Hz/point) and DAY (0.0063 Hz/day) into relation, one point on the EDSS scale would correspond to approx. 52 days (in a linear model). We tested against a hypothesized change of zero, which was a conservative scenario. If finger tapping would improve without rehabilitation, we would see a positive correlation between age and tapping performance and if tapping frequencies would stay untouched by MS, the association with the EDSS would not be visible. We therefore assume that finger tapping can display a sensorimotor dimension of impairment by MS and that changes during rehabilitation represent a counteract to those impairments. Further, it is important to keep in mind that SESSION was examining potential learning effects of the task, while DAY was examining if it was beneficial to have more days of rehabilitation.

The impact of both EDSS and the interaction of EDSS and LAT suggests that although a lateralization can show up in a severe way (we observed a maximum difference of 57% of the better performing hand between both hands in one patient), the strongest impairment can in general be expected global. However, if the motor capacity of one body-side low-passes a certain threshold for daily functioning, it could be experienced as a strongly body-side emphasized MS symptomatology. Based on this, we strongly recommend to always include both body-sides in assessments, even when one is reported as being non-pathological.

As observed in prior work^20^, age and MS can both impair certain aspects of sensorimotor control, with age usually appearing as a mediator (the association of EDSS and sensorimotor performance was in almost all tests attenuated by age). However, age was observed to still be a moderate to strong performance factor; one potential explanation that we were not able to see an impact of age in our study could be the relatively low maximum age of our participants, as it has been shown that age can affect sensorimotor performance in a non-linear way, with an “excess” at the end of the seventh decade^36^.

The model for STATE revealed noteworthy peculiarities: The reported state, the way the patients estimated their day form, was not only not associated with their task performance, but was to some extend the result of the trait MS (by EDSS) and to a quite small proportion the time of the day. While the EDSS was associated with the tapping performance, the time of day revealed no significant impact in the TAP model. Further, we did not observe a significant impact of DAY on STATE. We conclude that there was apparently no strong influence of rehabilitation on the experienced day form. This could either be due quick adaptations of expectations (the “normal state” to compare with) or due to a question that appears unspecific^37^. STATE could therefore be rather a trait than a state and could potentially be neglected in such an assessment. We do not know if this is generalizable to, for instance, gait, balance, or other functions; although a study by Morris et al.^21^ reporting no association between experienced fatigue and walking performance would indicate that this could be possible, same as in Kalron and Aloni^38^, where depressive symptoms were associated with self-perception of walking ability, but not with quantitative gait parameters. According to our assumptions, the EDSS would be the intercept of STATE (the day-to-day fluctuations of the EDSS should be close to zero). Since TIME only accounted for a very small amount of variance, it could potentially be neglected outside the laboratory. The large amount of variance explained by SUBJECT would further emphasize character traits as candidates to explain the general tendency of a patient’s feelings. A review on cognitive dysfunction and sleep disturbances in MS also showed that subjective measures are rather associated with other subjective measures and, vice versa, objective measures are associated with objective measures^39^. The observed independence of this assessment should not indicate to ignore the (emotional) state of a patient in general, but should rather offer a tool to quickly assess a surrogate of the current sensorimotor capacity without the necessity to correct for this dimension (STATE). For instance, we do not know, if a maximum test over 20 s or 30 s would have had a comparable result.

A few limitations of the current study have to be mentioned. First, the sample size of 40 patients was relatively small, so weaker factors did not reveal significance (e.g., biological sex and age^15–17^). A post-hoc power analysis, however, did reveal good power estimates for the main effects of the TAP model (EDSS and HAND). Further, we did not differentiate between different forms of MS (relapsing remitting versus progressive forms), although there is some evidence suggesting a potential impact^19^; unfortunately, it remains unclear if they had comparable EDSS scores in the remitting and progressive samples (in Ref.^19^). Time spans between and time points of session were not fully controlled by the study group and strongly dictated by the participants’ schedules, however, resulting distributions appeared to be sufficient for the statistical approach. Another limitation is that we were not able to test every factor suggested by literature, like training^17^ or arousal^22^, and do not have information on medication and changes of its prescription. Also, we do not know, to what extent tapping performance is associated with performance in activities of daily living. However, evidence from stroke would suggest this^40^. As mentioned above, it remains unclear if our findings on STATE could be generalized to other tasks (for instance walking tests or reaction time tasks), although there is some evidence supporting this^22^. Further, we treated a potentially ordinal variable (STATE) as continuous, based on its normal distribution and common use as a continuous variable^41^. We tried to circumvent this problem by a simulation and the results suggested that the initial findings appear to be reliable. Lastly, we did not cover all potential factors that are displayed in Fig. 1. Especially between-subject factors like training or relative strength were not covered and could be able to explain part of the subject variance in the model.

## Conclusion

The tapping test, using the whole upper-limb over 10 s, proved to be a valid, reliable, and feasible assessment tool for MS patients and could be used as a quick assessment for screening purposes, in experiments with repeated measures, or as an additional measure of upper-limb function in future studies on disease progression or interventions (e.g., movement therapy or medication). In our sample of 40 MS patients, we were not able to identify other additional factors (additional to the EDSS) than hand-dominance and symptom laterality of MS, and were further able to show a positive impact of neurologic inpatient rehabilitation on task performance. The reported day form of patients was not associated with performance and rather reflected disease severity and the time of the day.

## Data Availability

On request.
